# Neuroprotective effects of arctigenin on cerebral ischemia-reperfusion injury in rats via the EPO/EPOR-JAK2-STAT5 signaling pathway

**DOI:** 10.3389/fphar.2025.1503971

**Published:** 2025-03-26

**Authors:** Shanshan Xu, Yuting Chen, Lingling Zhang, Wei Lu, Xu Chen, Ting Wang, Wenjie Wang

**Affiliations:** ^1^ Department of Pharmacy, Taizhou Central Hospital (Taizhou University Hospital), Taizhou University, Taizhou, China; ^2^ College of Pharmacy, Liaoning University of Traditional Chinese Medicine, Shenyang, China; ^3^ School of Medicine, Taizhou University, Taizhou, Zhejiang, China

**Keywords:** arctigenin, cerebral ischemia-reperfusion injury, EPO, JAK2/STAT5, EPOR

## Abstract

**Introduction:**

Cerebral ischemia-reperfusion injury (CIRI) is a complex pathophysiological process with significant morbidity and mortality, and there is no specific agent. Previous studies have found that arctigenin can play an anti-CIRI role through anti-inflammatory and antioxidant effects. This study further explored the anti-CIRI mechanism of arctigenin via the EPO/EPOR-JAK2-STAT5 signaling pathway.

**Methods:**

TTC and H&E staining were used to observe infarct volume and morphological changes in the brain, RT-PCR was used to detect EPO, EPOR, HIF, JAK2, STAT5, NF-κB mRNA expression, EPO/EPOR ratio was detected by immunofluorescence, and HIF was observed by immunohistochemical staining. The protein expression levels of JAK2 and STAT5 were detected, and the protein expression levels of EPO, EPOR, HIF, JAK2 and STAT5 were detected by western blot.

**Results:**

Our results indicate that arctigenin significantly reduced infarct volume and improved histopathological changes in the brain tissues from CIRI rats at 24 h, 48 h, and 72 h after reperfusion by TTC and H&E staining. RT-PCR analysis showed that arctigenin could significantly upregulate the mRNA expressions of EPO, EPOR, and HIF and downregulate the mRNA expressions of JAK2, STAT5, and NF-κB in the brain tissues from CIRI rats at 24 h, 48 h, and 72 h after reperfusion. Immunofluorescence assay showed that the ratio of EPO/EPOR in CIRI rats at 24 h, 48 h, and 72 h post-reperfusion was significantly elevated by arctigenin. Arctigenin could upregulate the HIF protein expression while downregulate the protein expressions of JAK2, STAT5, and NFκB in the brain tissues from CIRI rats at 24 h, 48 h, and 72 h after reperfusion by immunohistochemical staining. The protein regulation results of EPO, EPOR, HIF, JAK2, and STAT5 were also confirmed by Western blot at 72 h after reperfusion, consistent with the above results.

**Discussion:**

In conclusion, arctigenin demonstrated neuroprotective properties against CIRI potentially through the EPO/EPOR-JAK2-STAT5 signaling pathway. These findings provide a scientific rationale for further exploration of arctigenin as a therapeutic agent for stroke.

## Highlights

1. Arctigenin reduced infarction volume and improved brain tissue in CIRI rats.
2. Neuroprotection of arctigenin may operate through the EPO/EPOR-JAK2-STAT5 pathway.
3. Arctigenin inhibiting inflammation and neuro-damage in CIRI rats.
4. The study supports arctigenin as a neuroprotective for stroke treatment.

## 1 Introduction

Stroke is a significant cause of disability and death worldwide. As the population ages, stroke has emerged as the leading cause of death and adult disability among our residents. Acute ischemic stroke, which accounts for approximately 80% of all stroke cases, is particularly concerning (Powers, 2020; Tsai et al., 2024). It places a heavy medical, economic, and social burden on the country, its patients, and their families (Lucas-Noll et al., 2023). The brain is one of the most essential organs in the human body. It is susceptible to ischemia (lack of blood flow) and hypoxia (lack of oxygen) and is highly vulnerable to ischemic damage. The resulting tissue damage and dysfunction can worsen if blood flow is restored after cerebral ischemia. This can even lead to irreversible injury, a condition known as cerebral ischemia-reperfusion injury (Ni et al., 2022). Cerebral ischemia-reperfusion is a critical pathophysiological process in ischemic stroke. Effectively preventing and treating cerebral ischemia-reperfusion injury is a key topic in the medical field.

The cerebral ischemia-reperfusion pathological process involved excitatory amino acid toxicity, inflammatory response, intracellular Ca2+ overload, oxidative stress, blood-brain barrier destruction, and neuronal cell apoptosis (Li et al., 2024). Numerous experimental studies have shown that Erythropoietin (EPO) and its receptor EPOR from mature neurons and glial cells play an important role in regulating the growth and development of the nervous system (Wang et al., 2017; Wang et al., 2022). EPO/EPOR might be involved in the endogenous protection of nerve cells against harmful stimulation damage to the central nervous system (Tanaka and Nangaku, 2012). Under hypoxia, hypoxia-inducible factor-l (HIF-l) could promote the expression and release of brain-derived EPO and play a protective role (Zhang et al., 2021). Meanwhile, EPO/EPOR could activate downstream signaling pathways, including phosphatidylinositol 3-kinase (PI3K), Janus kinase 2 (JAK2) and signal transducer and activator of transcription 5 (STAT5), and other pathways to participate in apoptosis, excitotoxicity, inflammation, oxidative stress, and other physiological processes; Among them, JAK2/STAT5 is the main signal transduction pathway of EPO against neuronal cell apoptosis after cerebral ischemia (Lv et al., 2017; Sergio and Rolando, 2022).

Arctigenin is one of the main pharmacologically active ingredients of dry, mature fruit from Arctium lappa L. Studies have shown that arctigenin can protect against brain damage through anti-inflammation and anti-apoptosis (Song et al., 2016). Its anti-inflammatory activity is due to the inhibition of the JAK-STAT pathway (Kou et al., 2011). Our previous study found that arctigenin has an antioxidant effect, which can significantly improve the SOD content, reduce the content of LDH and MDA in brain tissue, inhibit the production of oxygen free radicals, protect the brain damage caused by cerebral ischemia and reperfusion, and can achieve anti-inflammatory effect by inhibiting the secretion of IL-1 β, IL-6, and TNF-α in the ischemic penumbra (Xu et al., 2020). However, the mechanism of arctigenin has not been thoroughly studied. The study aims to investigate the protective effect of arctigenin on ischemic reperfusion injury based on the EPO/EPOR-JAK2-STAT5 signal transduction pathway.

## 2 Materials and methods

### 2.1 Materials

#### 2.1.1 Animals

A total of 140 SPF female SD rats, weighing 300 ± 20g, were purchased from Beijing Vital River Laboratory Animal Technology Co., Ltd. (License Number: SCXK (Jing) 2012-0001). They were fed a standard pellet diet and had free access to water, with a 12-hour light-dark cycle. After purchase, the rats were acclimated for 1 week in the SPF animal room. Ethical approval was obtained by Taizhou University (No. TZXY-2022-20221020).

#### 2.1.2 Reagents

The radix of Arctium lappa (Batch number: 18070901) was obtained from Taizhou Fuda Traditional Chinese Medicine Pieces Co., Ltd., Zhejiang Province. The arctigenin was prepared in the laboratory according to reference (Guan et al., 2015), using acid hydrolysis and alcohol extraction from the radix of Arctium lappa. The crude product with a purity >75% was obtained by ethyl acetate extraction and crystallization with ethanol to yield pure arctigenin. Its purity was further confirmed to be ≥ 98% by high-performance liquid chromatography [HPLC, (250 mm × 4.6 mm, 5 μm) Waters-C18 column, column temperature 30 °C, mobile phase consisting of acetonitrile-water (37:63), detection wavelength at 283 nm]. RIPA cell lysis buffer (C1053, Beijing Applygen Technologies Inc., Beijing, China), BCA Protein Assay Kit (CW0014S, CWBIO, Beijing, China), 30% acrylamide (PAGE Pre-Solution) (A1010, Solarbio, Beijing, China), 1M Tris-HCl buffer solution (pH = 6.8) (T1020, Solarbio, Beijing, China), 1.5M Tris-HCl buffer solution (pH = 8.8) (T1010, Solarbio, Beijing, China), anti-HIF-1 antibody (ab51608, Abcam, USA), anti-JAK2 antibody (ab32101, Abcam, USA), anti-STAT5 antibody (ab230670, Abcam, USA), anti-EPO antibody (ab226956, Abcam, USA), anti-EPO-R antibody (ab244202, Abcam, United States), anti-β-actin antibody (ab8226, Abcam, USA), FITC-labeled goat anti-mouse IgG (H+L) (A0568, Beyotime Biotechnology, Shanghai, China), Cy3-labeled goat anti-rabbit IgG (H+L) (A0516, Beyotime Biotechnology, Shanghai, China), horseradish peroxidase-labeled goat anti-rabbit IgG (H+L) (ZB-2301, Beijing Zhongshan Jinqiao Biotechnology Co., Ltd, Beijing, China), horseradish peroxidase-labeled goat anti-mouse IgG (H+L) (ZB-2305, Beijing Zhongshan Jinqiao Biotechnology Co., Ltd, Beijing, China), SYBR®Green Realtime PCR Master Mix (QPK-201, Toyobo, Japan).

### 2.2 Methods

#### 2.2.1 Animal experiments

Firstly, 140 rats were randomly divided into 5 groups: control group, sham group, model group, EPO antibody group, and arctigenin group (100 mg/kg), with 28 rats in each group. The arctigenin group received corresponding doses of arctigenin. Rats in the control group, sham group, model group, and EPO antibody group were orally administered the same dose of saline at 0.01 mL/g once a day for 2 weeks. Following the final administration, a modified line embolization technique was employed to establish a rat model of cerebral ischemia and reperfusion, which included the model group, the EPO antibody group, and the arctigenin group. The modeling approach was based on relevant literature (Yao et al., 2023). Rats in the sham group underwent the same procedure, but the depth of insertion of the fishing line was only 0.5–1 cm, causing no substantial occlusion to the rats. The EPO antibody group received intravenous an injection of rabbit anti-rat EPO antibody via the tail vein, at a dose of 50 μL/kg, once daily thereafter: once for the 24-hour group, twice for the 48-hour group, and three times for the 72-hour group. Twelve rats from each group were randomly selected for euthanasia and specimen collection at 24, 48, and 72 h post-reperfusion, respectively. Thirty minutes after the final administration, rats were anesthetized with pentobarbital sodium, positioned supine on the operating table, decapitated, and brains were dissected to isolate ischemic penumbra brain tissue (corresponding areas of cerebral cortex for the control group and sham group), which were then stored at −80°C for future analysis.

#### 2.2.2 TTC staining and measurement of infarct volume

After modeling, several rats from each group were randomly selected and euthanized using excess anesthesia, decapitated, and brains were harvested. The whole brains were placed on an ice tray, frozen at −20°C for 30 min, removed, placed on an ice surface, and sectioned coronally from the frontal pole to the cerebellum into slices with a thickness of 2 mm. The brain slices were immersed in 2% 2,3,5-triphenyl tetrazolium chloride (TTC) solution and incubated at 37°C for 30 min, with flipping every 15 min. After incubation, the brain slices were removed and fixed in a 10% formaldehyde solution to observe the presence and location of infarcts, and photographs were taken. An image analysis system was used to obtain the area of infarcts on each slice and then calculate the volume.

#### 2.2.3 Histological analysis

Fresh brain tissue is fixed in 4% paraformaldehyde for over 24 h. Once fixation is complete, the tissue is removed from the fixative solution, and the desired area is expertly trimmed with a scalpel in a fume hood. The trimmed tissue, accompanied by its corresponding label, is placed in a dehydration box for the dehydration, embedding, and sectioning processes. The paraffin sections are then sequentially immersed in the following solutions: xylene I for 20 min, xylene II for 20 min, absolute ethanol I for 10 min, absolute ethanol II for 10 min, 95% ethanol for 5 min, 80% ethanol for 5 min, and 70% ethanol for 5 min, followed by a wash with distilled water. Finally, the sections are stained with hematoxylin to highlight the nuclei and eosin to stain the cytoplasm, concluding with dehydration and mounting. An optical microscope (CX33, Olympus, Tokyo, Japan) was used to examine the sections at a magnification of 400.

#### 2.2.4 RT-PCR analysis

Fresh brain tissue was obtained, and RNA was extracted and quantified for concentration. cDNA synthesis was performed through reverse transcription, followed by amplification using a specialized machine. As previously described (Chen et al., 2021), RT-PCR analysis was carried out to evaluate the expression levels of EPO, EPOR, HIF-1, JAK2, STAT5, and NF-κB. This analysis was conducted using SYBR^®^ Green Realtime PCR Master Mix, following the manufacturer’s protocol. The primers were as follows: EPOR forward: 5′-TGT​TTC​TGG​GAG​GAA​GCG​GCG​AAC​T-3′ and reverse: 5′-ATG​GAT​GAT​GCG​GTG​GTA​GCG​AGG​AG-3’; EPO forward: 5′-CCC​TGC​TGC​TTT​TAC​TA-3′ and reverse: 5′-ACA​TTT​TCT​GCC​TCC​TT-3’; HIF-1 forward: 5′-AGA​ACT​CTC​AGC​CAC​AGT​GC-3′ and reverse: 5′-CTA​GCA​GAG​TCA​GGG​CAT​CG-3’; JAK2 forward: 5′- TTG​TGG​TAT​TAC​GCC​TGT​GTA​TC-3′ and reverse: 5′-ATG​CCT​GGT​TGA​CTC​GTC​TAT-3’; STAT5 forward: 5′- GCT​GGA​AGC​CTT​GCT​GAT-3′ and reverse: 5′-TCC​TCA​AAC​GTC​TGG​TTG​ATC-3’; β-actin forward: 5′-AGG​GAA​ATC​GTG​CGT​GAC-3′ and reverse: 5′-CAT​ACC​CAA​GAA​GGA​AGG​CT-3’. The mRNAs expressions were determined by the 2^−ΔΔCT^ method.

#### 2.2.5 Immunofluorescence assay

Immunofluorescence (IF) was performed to evaluate the ratio of EPO/EPOR according to the reference (Luo et al., 2023). Briefly, the fixed brain tissue was sectioned, deparaffinized, and rehydrated. Then, the brain sections (10 μm) were blocked, incubated with anti-EPO antibody and anti-EPOR antibody at 4°C overnight, and subsequently incubated with PE- and FITC-conjugated secondary antibody at room temperature for 1h, respectively. DAPI was used to visualize cell nuclei. The fluorescence microscope was used to visualize and capture the fluorescence signal indicating the presence and location of EPO and EPOR.

#### 2.2.6 Immunohistochemical staining

The brain paraffin sections were also used in the immunohistochemical staining analysis to evaluate the expression of HIF-1, JAK2, STAT5, and NF-κB, according to the previous study (Chen et al., 2021). According to the manufacturer’s instructions, immunohistochemistry was performed using a Dako En Vision System (Dako Diagnostics AG Zug, Switzerland). An Olympus microscope was used to acquire the images. Image-Pro-Plus version 6.0 software (Media Cybernetics, Inc., Rockville, MD, USA) was used to evaluate the area and density of the stained region, along with the integrated optical density (IOD) value of the immunohistochemistry section. Densitometry analysis of the digital image was conducted to assess the average level of protein staining at ×400 magnification. The quantification of signal density was performed in a blinded manner across five randomly selected tissue regions, followed by subsequent statistical analysis.

#### 2.2.7 Western blot analysis

Western blot analysis was executed following the procedures outlined in previous literature (Xu et al., 2024). In summary, tissue samples were excised and lysed to extract the supernatant, which was subsequently quantified using a BCA protein assay kit. Following this, the samples were resolved and transferred to PVDF membranes, which were then incubated with primary antibodies, followed by secondary antibodies. Subsequently, the ECL (New Cell & Molecular Biotech Co., Ltd) method was used to visualize, and the gel imaging analysis system (Tanon 4600, Shanghai Tanon Science & Technology Co., Ltd) was used to analyze. The intensity of each protein band was measured by Image-Pro Plus 6.0 software (Media Cybernetics, Inc., Rockville, MD, United States) with GAPDH as the endogenous control.

### 2.3 Statistical analysis

Data were presented as mean ± standard deviation. One-way analysis of variance (ANOVA) was conducted using SPSS 20.0 (Chicago, IL, United States) software to analyze inter-group differences. *p* < 0.05 is considered as statistically significant.

## 3 Results

### 3.1 Effect of arctigenin on cerebral infarction volume of brain in cerebral ischemia-reperfusion model rats

**FIGURE 1:**
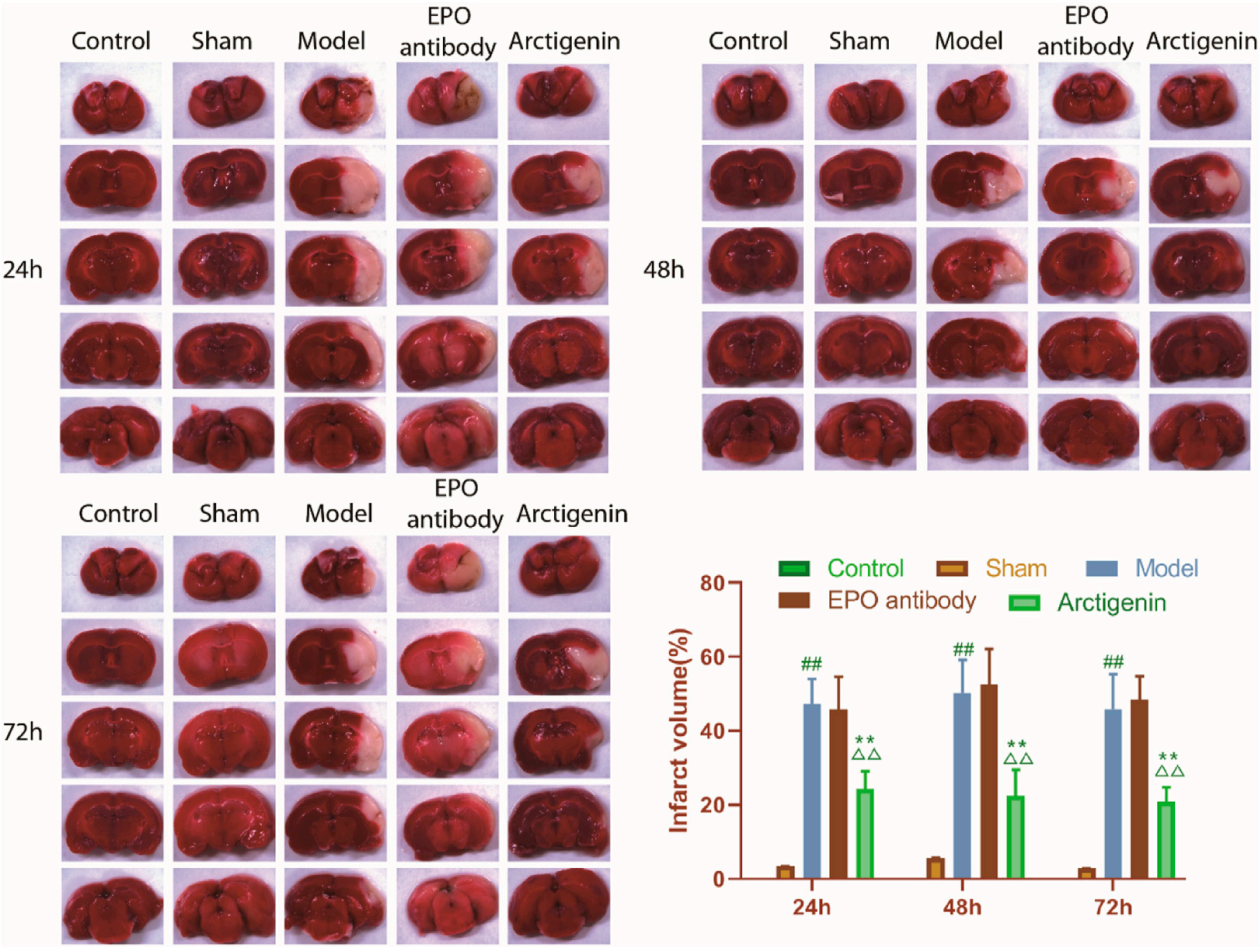
Effect of arctigenin on cerebral infarction volume in cerebral ischemia-reperfusion model rats. Compared with sham group, ^##^p < 0.01; Compared with model group, **p < 0.01; Compared with EPO antibody group, ^△△^p < 0.01.

After TTC staining, the non-infarcted area appeared bright red, while the infarcted area appeared pale. In the control group and sham group, brain tissues were stained bright red without pale infarcted areas. Different degrees of infarction were observed at 24 h, 48 h, and 72 h after reperfusion in the remaining groups. The model group showed significantly pale infarcted tissues compared to the sham group (p < 0.01). There was no significant difference between the EPO antibody group and the model group (p > 0.05). Compared with the model group and the EPO antibody group, the arctigenin group showed a significantly reduced infarction volume (p < 0.01), indicating that arctigenin can alleviate the damage after ischemia/reperfusion in rats (Figure 1).

### 3.2 Effect of arctigenin on the pathology of brain in cerebral ischemia-reperfusion model rats

**FIGURE 2:**
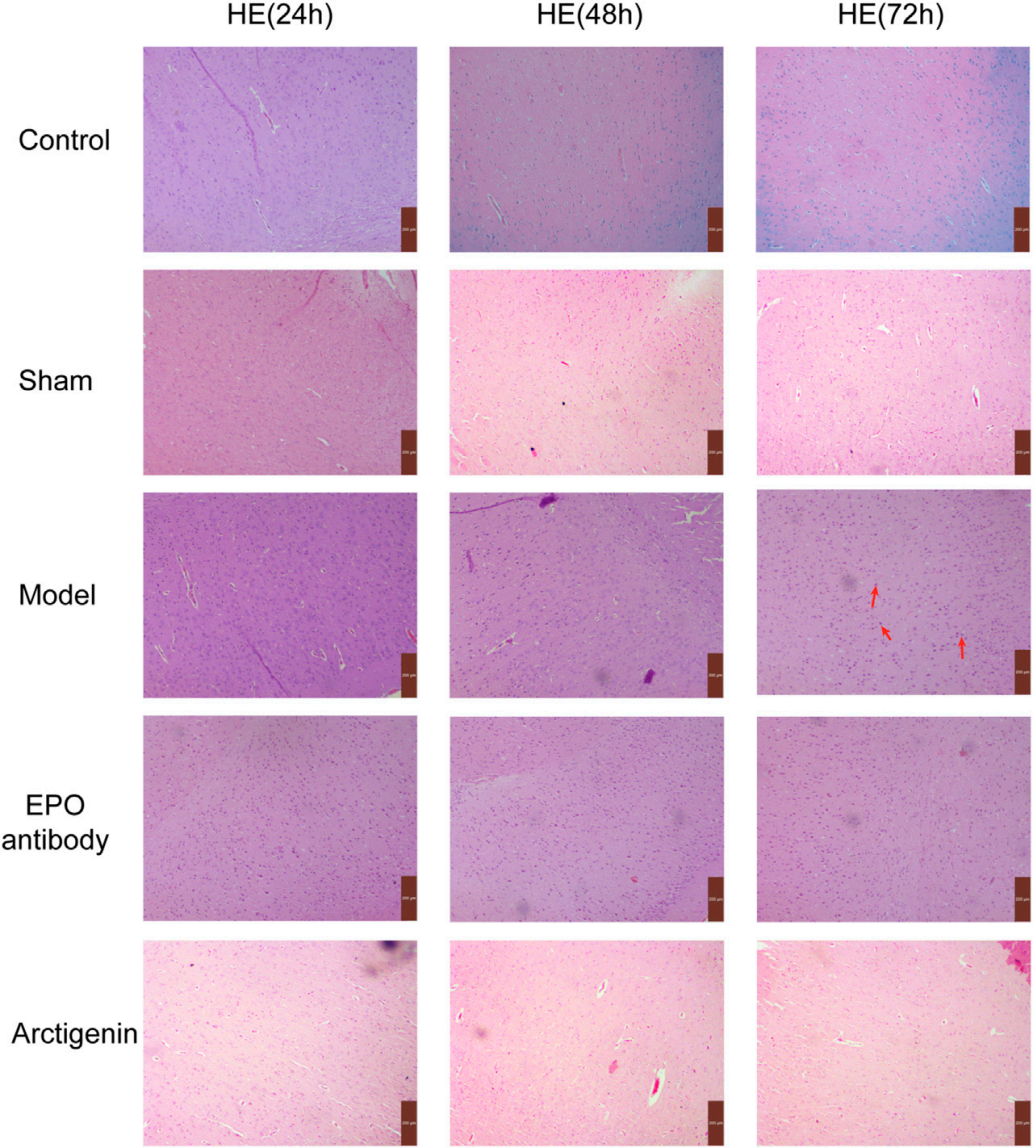
Histopathology of ischemic brain in each group (×200).

In the control and sham groups, neuronal cells in brain tissue were primarily intact, with clear outlines, regular-shaped nuclei located centrally within the cells, and cytoplasm-stained light blue, showing no significant pathological changes. In the model and EPO antibody group, cells exhibited irregular arrangement, noticeable morphological changes, nuclear fragmentation, and alignment towards the cell periphery, accompanied by interstitial edema and inflammatory cell infiltration. There is also some neuronal degeneration, as shown by the red arrows in the figure. While treatment with arctigenin, the neuronal cell damage was significantly alleviated (Figure 2).

### 3.3 The effects of arctigenin on the mRNA expression of EPO, EPOR, HIF-1, JAK2, STAT5, and NF-κB in the cerebral ischemia-reperfusion model rats

**FIGURE 3:**
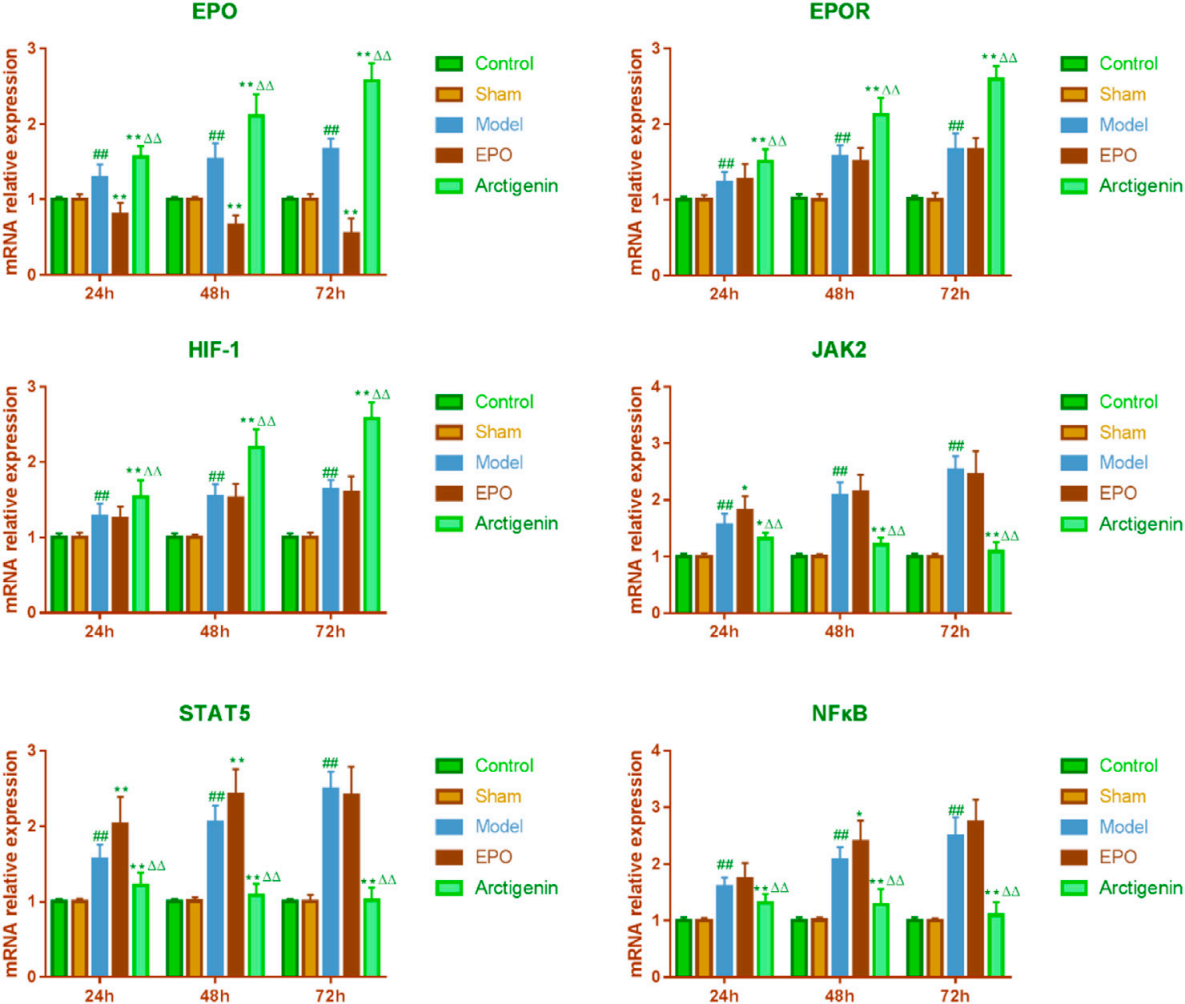
The effects of arctigenin on the mRNA expression of HIF-1, JAK2, STAT5, and NF-κB in the cerebral ischemia-reperfusion model rats by IHC method. Compared to the sham group, ^##^p < 0.01; compared to the model group, *p < 0.01, **p < 0.01; compared to the EPO antibody group, ^△△^p < 0.01.

Compared to the sham group, the EPO and EPOR mRNA expressions in the ischemic penumbra of brain tissue in the reperfusion model group rats were significantly increased at 24 h, 48 h, and 72 h post-reperfusion (p < 0.01). In contrast to the model group, the EPO mRNA expression in the EPO antibody group was significantly decreased at 24 h, 48 h, and 72 h post-reperfusion (p < 0.01), while the EPOR mRNA expression showed no significant difference (p > 0.05). Compared to the model and EPO antibody groups, the mRNA expressions of EPO and EPOR in the arctigenin group were significantly increased at the same time points post-reperfusion (p < 0.01). Similarly, the HIF-1 mRNA expression in the ischemic penumbra of brain tissue in the reperfusion model group rats was significantly elevated at 24 h, 48 h, and 72 h post-reperfusion compared to the sham group (p < 0.01). There was no significant difference in the HIF-1 mRNA expression between the EPO antibody group and the model group at 24 h, 48 h, and 72 h post-reperfusion. However, the HIF-1 mRNA expression in the arctigenin group was significantly increased at 24 h, 48 h, and 72 h post-reperfusion compared to both the model and EPO antibody groups (p < 0.01). The JAK2 mRNA expression in the ischemic penumbra of brain tissue in the model group rats was significantly increased at 24h, 48h, and 72 h post-reperfusion compared to the sham group (p < 0.01). In comparison to the model group, the JAK2 mRNA expression in the EPO antibody group was increased at 24 h (p < 0.05), with no significant difference observed at 48 h and 72 h post-reperfusion (p > 0.05). In the arctigenin group, the JAK2 mRNA expression was decreased at 24 h, 48 h, and 72 h post-reperfusion (p < 0.05 or p < 0.01) compared to the model group and EPO antibody group. In comparison to the sham group, the STAT5 mRNA expression in the ischemic penumbra of brain tissue in the model group rats was significantly increased at 24 h, 48 h, and 72 h post-reperfusion (p < 0.01). Compared to the model group, the STAT5 mRNA expression in the EPO antibody group was significantly increased at 24 h and 48 h post-reperfusion (p < 0.01), with no significant difference observed at 72 h (p > 0.05). In contrast, the STAT5 mRNA expression in the arctigenin group was significantly decreased at 24 h, 48 h, and 72 h post-reperfusion compared to both the model and EPO antibody groups (p < 0.01). Lastly, the NF-κB mRNA expression in the ischemic penumbra of brain tissue in the model group rats was significantly higher at 24 h, 48 h, and 72 h post-reperfusion compared to the sham group (p < 0.01). In comparison to the model group, there was no significant difference in the NF-κB mRNA expression in the EPO antibody group at 24 h and 72 h post-reperfusion, but it was increased at 48 h (p < 0.05). However, the NF-κB mRNA expression in the arctigenin group was significantly decreased at 24 h, 48 h, and 72 h post-reperfusion compared to both the model and EPO antibody groups (p < 0.01) (Figure 3).

### 3.4 Effects of arctigenin on the ratio of EPO/EPOR in the cerebral ischemia-reperfusion model rats

**FIGURE 4:**
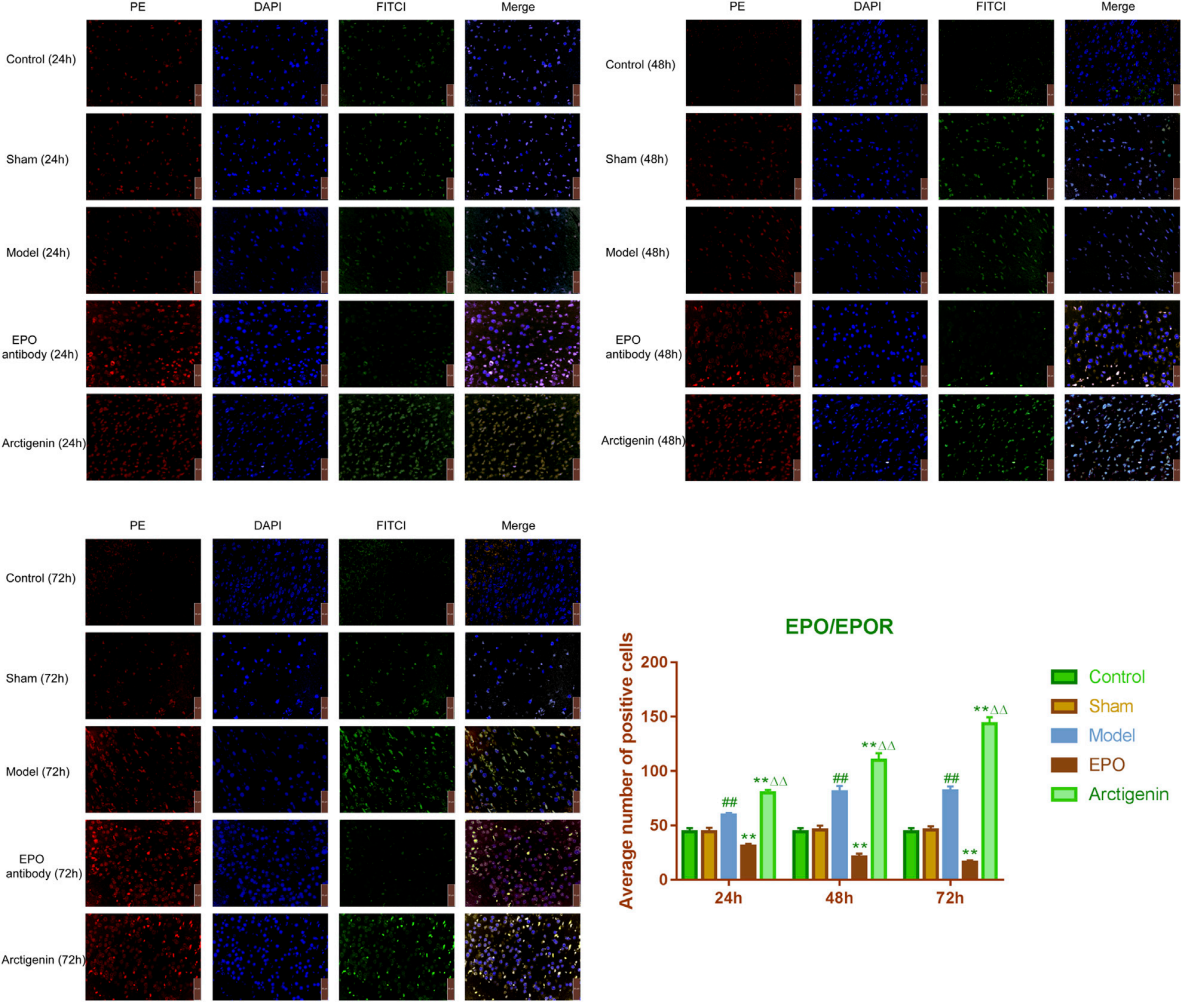
The effect of arctigenin on the ratio of EPO/EPOR in the cerebral ischemia-reperfusion model rats (bar = 50 µm). Compared to the sham group, ^##^P<0.01; compared to the model group, **P < 0.01; compared to the EPO antibody group, ^△△^P < 0.01.

In comparison to the sham group, the EPO/EPOR ratio in the ischemic penumbra of brain tissue from the model group rats was significantly elevated at 24, 48, and 72 h post-reperfusion (p < 0.01). When contrasted with the model group, the EPO/EPOR ratio in the EPO antibody group showed a significant reduction at the same time points (p < 0.01). Additionally, the arctigenin group exhibited a significantly higher EPO/EPOR ratio at 24, 48, and 72 h post-reperfusion compared to both the model group and the EPO antibody group (p < 0.01) (Figure 4).

### 3.5 The effects of arctigenin on the protein expression of HIF-1, JAK2, STAT5, and NF-κB in the cerebral ischemia-reperfusion model rats by IHC method

**FIGURE 5:**
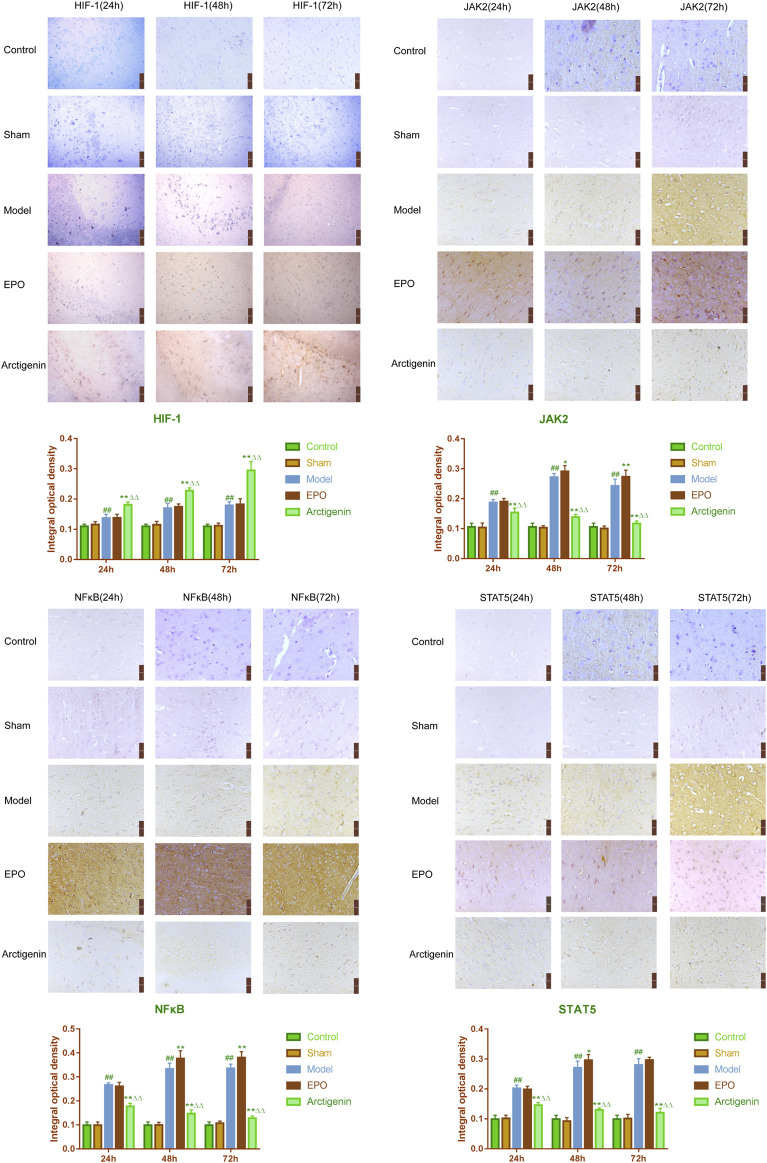
The effects of arctigenin on the protein expression of HIF-1, JAK2, STAT5, and NF-κB in the cerebral ischemia-reperfusion model rats by IHC method (bar = 50 µm). Compared to the sham group, ^##^p < 0.01; compared to the model group, *p < 0.01, **p < 0.01; compared to the EPO antibody group, ^△△^p < 0.01.

In comparison to the sham group, the protein expression of HIF-1 in the ischemic penumbra of the brain tissue in the model group rats demonstrated a significant upregulation at 24, 48, and 72 h following reperfusion (p < 0.01). No statistically significant difference was observed between the model group rats and the EPO antibody group at the 24, 48, and 72 h post-reperfusion time points (p > 0.05). In contrast, the expression of HIF-1 protein in the arctigenin group at these same time intervals post-reperfusion was significantly elevated when compared to both the model and EPO antibody groups (p < 0.01). JAK2 was significantly elevated in the ischemic penumbra of brain tissue in the model group rats at 24h, 48h, and 72 h post-reperfusion compared to the sham group (p < 0.01). In contrast to the model group, the JAK2 protein expression in the EPO antibody group showed no significant difference at 24 h post-reperfusion (p > 0.05), while it was increased at 48 h (p < 0.05) and significantly increased at 72 h (p < 0.01). The JAK2 protein expression in the arctigenin group at 24 h, 48 h, and 72 h post-reperfusion significantly decreased compared to the model and EPO antibody groups (p < 0.01). Similarly, the STAT5 protein expression in the ischemic penumbra of brain tissue in the model group rats was significantly increased at 24 h, 48 h, and 72 h post-reperfusion compared to the sham group (p < 0.01). There was no significant difference in STAT5 protein expression between the EPO antibody and model groups at 24 h and 72 h post-reperfusion (p > 0.05). Still, an increase was observed in the EPO antibody group at 48 h (p < 0.05). The STAT5 protein expression in the arctigenin group at 24 h, 48 h, and 72 h post-reperfusion was significantly reduced compared to the model and EPO antibody groups (p < 0.01). The NF-κB protein expression in the ischemic penumbra of brain tissue in the model group rats was significantly upregulated at 24 h, 48 h, and 72 h post-reperfusion compared to the sham group (p < 0.01). Compared to the model group, the NF-κB protein expression in the EPO antibody group showed no significant difference at 24 h post-reperfusion (p > 0.05). Still, it was significantly increased at 48h and 72 h (p < 0.01). The NF-κB protein expression in the arctigenin group at the corresponding time points post-reperfusion significantly decreased compared to the model and EPO antibody groups (p < 0.01) (Figure 5).

### 3.6 The effect of arctigenin on the protein expression of EPO, EPOR, HIF-1, JAK2, STAT5, and NF-κB in the cerebral ischemia-reperfusion model rats

**FIGURE 6:**
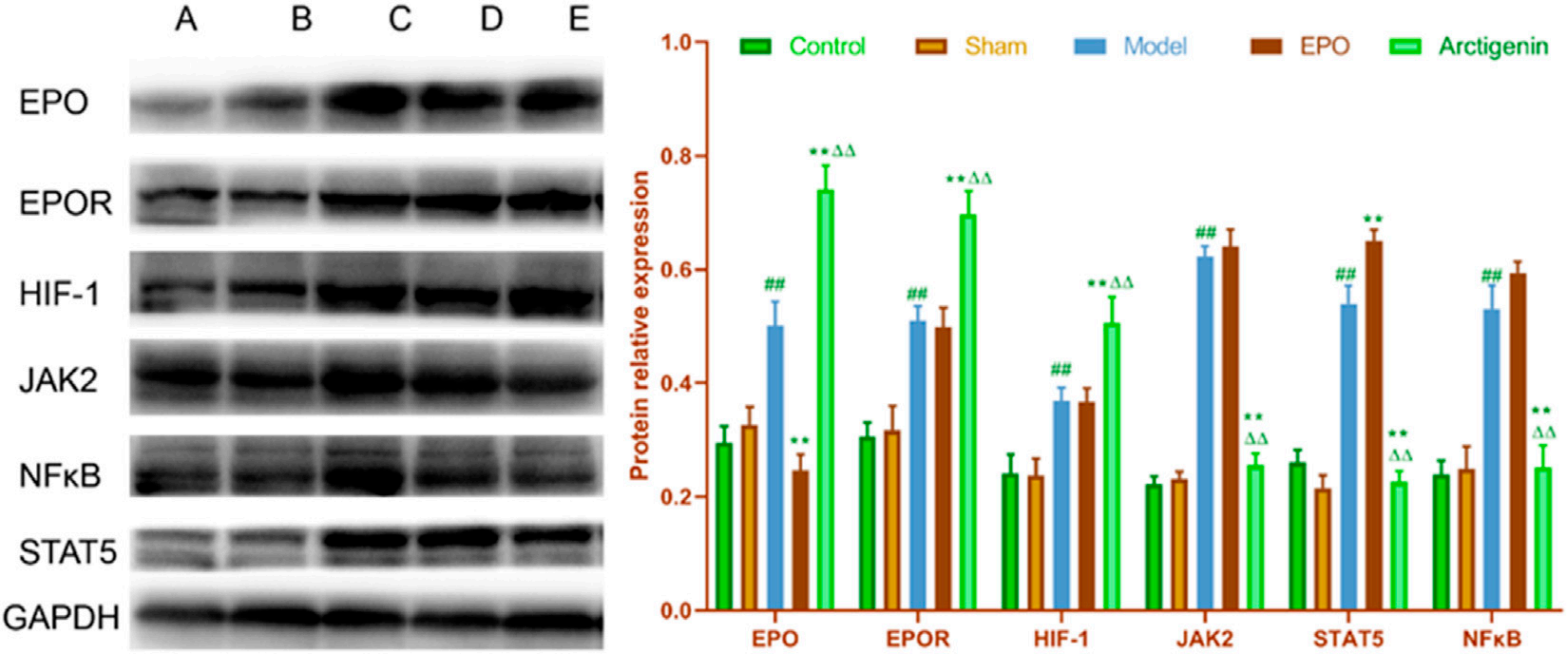
The effect of arctigenin on the protein expression of EPO, EPOR, HIF-1, JAK2, STAT5, and NF-κB in the cerebral ischemia-reperfusion model rats at 72 h post-reperfusion. Compared to the sham group, ^##^p < 0.01; compared to the model group, **p < 0.01; compared to the EPO antibody group, ^△△^p < 0.01. **(A)** Control group; **(B)** Sham group; **(C)** Model group; **(D)** EPO antibody group; **(E)** Arctigenin group.

Compared to the sham group, the protein expression of EPO and EPOR in the ischemic penumbra of brain tissue in the model group was significantly increased at 72 h post-reperfusion (p < 0.01). Compared to the model group, the EPO antibody group exhibited a significant decrease in EPO protein expression (p < 0.01), with no significant difference in EPOR protein expression (p > 0.05). In contrast, the arctigenin group showed a significant increase in EPO and EPOR protein expression compared to the model and EPO antibody groups (p < 0.01). Similarly, the HIF-1 protein expression in the ischemic penumbra of brain tissue was significantly elevated in the model group at 72 h post-reperfusion compared to the sham group (p < 0.01). There was no significant difference in HIF-1 protein expression between the EPO antibody and model groups (p > 0.05). However, the arctigenin group demonstrated a significant increase in HIF-1 content compared to the model and EPO antibody groups (p < 0.01). The JAK2 protein expression in the ischemic penumbra of brain tissue also showed a significant increase in the model group at 72 h post-reperfusion compared to the sham group (p < 0.01). Compared to the model group, the EPO antibody group did not show a significant difference in JAK2 protein expression (p > 0.05). In contrast, the arctigenin group significantly decreased JAK2 protein expression compared to the model and EPO antibody groups (p < 0.01). For STAT5 protein expression, a significant increase was observed in the ischemic penumbra of brain tissue in the model group at 72 h post-reperfusion compared to the sham group (p < 0.01). The EPO antibody group exhibited a significant increase in STAT5 protein expression at 72 h post-reperfusion compared to the model group (p < 0.01). Compared to the model and EPO antibody groups, the arctigenin group showed a significant decrease in STAT5 protein expression (p < 0.01). Lastly, the NF-κB protein expression in the ischemic penumbra of brain tissue was significantly higher in the model group at 72 h post-reperfusion compared to the sham group (p < 0.01). There was no significant difference in NF-κB protein expression between the EPO antibody and model groups (p > 0.01). However, the arctigenin group significantly decreased NF-κB protein expression compared to the model and EPO antibody groups (p < 0.01) (Figure 6).

## 4 Discussion

Cerebral ischemia-reperfusion injury was a significant complication of stroke, characterized by a multi-step and multi-pathway cascade of injury responses. The key elements involved in this complex regulatory network include inflammation, lipid peroxidation, oxidative stress, excitatory amino acid toxicity, apoptosis, intracellular calcium overload, mitochondrial failureand, and free radical damage, which can directly or indirectly lead to neuronal apoptosis and necrosis (Li et al., 2017; Liu et al., 2024; Suzuki et al., 2016). Inflammatory responses and oxidative stress were the primary causes of ischemia-reperfusion injury and had become a focal point in neuroscience research. Antioxidant strategies and reduction of inflammatory damage have emerged as one of the main avenues for treating ischemic cerebrovascular diseases. A previous study (Xu et al., 2020) confirmed the dual effects of arctigenin in antioxidation and anti-inflammation in the rat model of cerebral ischemia-reperfusion, but the depth mechanism had not been explored. In this study, we investigated the anti-ischemia reperfusion regulation effect of arctigenin based on the EPO/EPOR-JAK2-STAT5 pathway at different time points. Through TTC and HE staining, arctigenin could significantly reduce the cerebral infarction volume and inflammatory infiltration and significantly improve nerve injury at 24 h, 48 h, and 72 h after reperfusion, indicating that arctigenin has a good effect against cerebral ischemia-reperfusion, which also laid a good foundation for our subsequent mechanistic study.

HIF-1 is a nuclear transcription factor that mediates the cellular response to hypoxia (Grimm et al., 2005). Under hypoxic conditions, HIF-1 had been activated and further activated the transcription and expression of genes such as EPO and vascular endothelial growth factor, promoting cell survival and proliferation in low oxygen environments and exerting neuroprotective effects (Guo et al., 2019). Following cerebral hypoxia-ischemia, the upregulation of EPO and EPOR was involved in protecting ischemic neurons and promoting tissue regeneration (Siren et al., 2001). Studies have suggested that HIF-1 can alleviate neuronal apoptosis by upregulating EPO after ischemia in rat brains, which may represent a potential therapeutic approach for ischemic stroke (Li et al., 2020). After cardiopulmonary resuscitation, brain tissue damage due to ischemia-hypoxia occurred, and the expression of HIF-1 and downstream gene EPO in rat brain cells was upregulated, which might be an adaptive response of the body to global cerebral ischemia-hypoxia following cardiopulmonary resuscitation (Kang et al., 2013). The study also reported that in middle cerebral artery occlusion model rats, the protein expression levels of HIF-1 and EPO significantly increased, helping cells adapt to hypoxic conditions (Zhang et al., 2022). Our study showed that after cerebral ischemia-reperfusion, the expression of EPO, EPOR, and HIF-1 was enhanced, consistent with the trends reported in the literature. After intervention with arctigenin, the mRNA and protein expressions of EPO, EPOR, and HIF-1 in the ischemic penumbra of brain tissue were significantly increased, suggesting that arctigenin may increase the tolerance of brain cells to ischemia-hypoxia by upregulating the mRNA and protein expression of HIF-1, EPO/EPOR genes, thereby exerting neuroprotective effects in the brain.

The biological functions of EPO were mediated by its corresponding receptors on the surface of target cells. EPO exerted its functions by binding to EPOR, inducing the phosphorylation of JAK2, and activating the STAT5 pathway (Juenemann et al., 2020; Ma et al., 2022). Within the intracellular domain of EPOR, there were two highly conserved sequences, Box1 and Box2, which played a crucial role in mediating the activation of JAK2 by EPO. A single molecule of EPO could bind to two molecules of EPOR, changing its conformation to form a dimer, which triggered the phosphorylation and activation of adjacent JAK2 tyrosine kinase molecules. This further initiated downstream signal transduction pathways. Additionally, STATs were direct substrates of JAKs; activated JAK2 could induce the phosphorylation of C-terminal amino acid residues of STAT5 protein, leading to dimerization and translocation from the cytoplasm to the nucleus, where it activated the NF-κB, thereby exerting biological effects (Pan et al., 2017; Patel et al., 2021; Tothova et al., 2021; Wei et al., 2017). NF-κB is an essential gene that regulates the expression of anti-apoptotic genes during cellular activation and is present in nearly all cells, including neurons, glial cells, and cerebral vascular endothelial cells (Liu et al., 2019). Excessive activation of NF-κB was one of the mechanisms by which cerebral ischemia-reperfusion causes cell damage (Wu et al., 2021). The study found that in type 2 diabetic rats, the expression of inflammatory factors such as TNF-α, IL-1β, and NF-kB p65 protein increased after cerebral ischemia-reperfusion injury, suggesting that NF-κB may be involved in the damage caused by cerebral ischemia-reperfusion in diabetic rats (Liu et al., 2020). An injection of pegylated EPO (p-EPO) could reduce the activation level of NF-κB in middle cerebral artery occlusion mice, indicating that p-EPO can inhibit the activation of NF-κB and exert neuroprotective effects against cerebral ischemic injury through its antioxidant and anti-inflammatory properties (Im et al., 2020). Our study showed that after cerebral ischemia-reperfusion, the mRNA and protein expression of JAK2, STAT5, and NF-κB were enhanced. After intervention with arctigenin, the mRNA and protein expression of JAK2, STAT5, and NF-κB in the ischemic penumbra of brain tissue in rats at 24 h, 48 h, and 72 h post-ischemia-reperfusion was significantly reduced. These indicated that arctigenin could inhibit the inflammatory cascade mediated by the JAK2/STAT5/NF-κB pathway, reverse the effects induced by excessive activation of NF-κB, reduce the release of inflammatory factors, and decrease the inflammatory response, suggesting that arctigenin can significantly improve the neurofunctional damage in rats with cerebral ischemia-reperfusion.

In summary, cerebral ischemia-reperfusion injury is a complex pathophysiological process that involves the induction and activation of a series of gene transcriptions. Our study suggests that arctigenin exerts its protective effects against cerebral ischemia-reperfusion injury by upregulating the expression of EPO/EPOR and modulating the JAK2/STAT5 signal transduction pathway. The study could provide a necessary theoretical foundation for the treatment following stroke and for the further research and development of arctigenin.

## Data Availability

The datasets presented in this study can be found in online repositories. The names of the repository/repositories and accession number(s) can be found in the article/supplementary material.
